# Association of Race and Ethnicity With Stroke and Mortality Outcomes in Atrial Fibrillation

**DOI:** 10.1016/j.jacadv.2025.101860

**Published:** 2025-06-05

**Authors:** Utibe R. Essien, Jasmyn J. Tang, Nadejda Kim, Leslie R.M. Hausmann, Donna L. Washington, Maria K. Mor, Valerie P. Nguyen, Jared W. Magnani, Walid F. Gellad, Michael J. Fine

**Affiliations:** a Center for the Study of Healthcare Innovation, Implementation & Policy, Greater Los Angeles VA Healthcare System, Los Angeles, California, USA; b Division of General Internal Medicine and Health Services, David Geffen School of Medicine at the University of California Los Angeles, Los Angeles, California, USA; c Center for Health Equity Research and Promotion, VA Pittsburgh Healthcare System, Pittsburgh, Pennsylvania, USA; d Division of General Internal Medicine, University of Pittsburgh School of Medicine, Pittsburgh, Pennsylvania, USA; e Department of Biostatistics, Graduate School of Public Health, University of Pittsburgh, Pittsburgh, Pennsylvania, USA; f Department of Medicine, University of Pittsburgh School of Medicine, Pittsburgh, Pennsylvania, USA

**Keywords:** atrial fibrillation, ethnicity, mortality, race, stroke, veterans

## Abstract

**Background:**

Atrial fibrillation (AF) is associated with stroke and mortality. Research has demonstrated racial and ethnic disparities in AF outcomes, yet our understanding of the determinants of these disparities is limited.

**Objectives:**

The authors compared stroke and mortality by race and ethnicity for AF patients in the Veterans Health Administration.

**Methods:**

We identified Veterans Health Administration patients with incident AF from January 1, 2014, to December 31, 2021, with follow-up through May 31, 2022. Our independent variables were race (American Indian/Alaska Native (AI/AN), Asian, Black, and White) and ethnicity (Hispanic). Our primary outcomes were stroke and mortality incidence. Cox proportional hazard models assessed the association between race, ethnicity, and our outcomes, adjusting for sociodemographic, clinical, and facility factors.

**Results:**

Our cohort included 157,332 patients with AF; mean age 72.9 ± 10.5 years, 97.8% male and 2.2% female. Overall, 22,628 (14.7%) patients developed stroke (46.3 per 1,000 person-years), and 52,288 (33.2%) patients died (96.1 per 1,000 person-years). The adjusted HR (aHR) for stroke was higher for Black (aHR: 1.14; 95% CI: 1.09-1.20) than White patients, with no differences observed between AI/AN, Asian, or Hispanic and White patients. Mortality was lower for Asian (aHR: 0.85; 95% CI: 0.78-0.93), Black (aHR: 0.92; 95% CI: 0.89-0.95), and Hispanic (aHR: 0.82; 95% CI: 0.77-0.87) than White patients, with no difference observed for AI/AN patients.

**Conclusions:**

In a nationwide cohort of AF patients, we found significantly higher stroke rates for Black than White patients. Conversely, we observed significantly lower mortality rates for Asian, Black, and Hispanic patients than for White patients. Interventions to address factors associated with these disparities are essential.

Atrial fibrillation (AF) is the most common cardiac arrhythmia worldwide, affecting up to 6 million people in the United States alone.^1^, ^2^, ^3^ AF is associated with a higher risk of all-cause mortality and a 5-fold increased risk of stroke, which severely impacts functional status and quality of life.^4^^,^^5^ As the incidence of AF rises, so too have pharmacotherapies and procedures to help manage this condition, including oral anticoagulant therapy such as direct oral anticoagulants. These therapies have been shown to significantly reduce the risk of AF-related morbidity and mortality.^6^, ^7^, ^8^, ^9^, ^10^, ^11^ Yet, despite their availability, racial and ethnic disparities exist in the therapeutic management of patients with AF.^12^, ^13^, ^14^, ^15^

The gravity of these treatment disparities is amplified by the fact that minoritized patients (eg, American Indian, Asian, Black, and Hispanic) with AF have been shown to have higher risks of stroke and death than White individuals, and it remains unclear what factors drive these outcome differences.^13^^,^^14^^,^^16^^,^^17^ Some prior studies on racial and ethnic disparities in AF outcomes did not control for anticoagulant use or were conducted prior to U.S. Food and Drug Administration approval of direct oral anticoagulants and cardiovascular practice guidelines recommending this class of anticoagulants for stroke prevention in AF. Thus, how contemporary management of AF influences disparities in downstream AF outcomes remains understudied.^18^

The Veterans Health Administration (VA) oversees the largest integrated health system in the United States and provides patients with affordable inpatient, outpatient, and specialist care along with a low-cost, uniform national drug formulary.^19^ The VA also manages up to 1 million patients with AF.^20^ By largely eliminating the burden of the cost of care, the VA provides an ideal environment to investigate racial and ethnic health disparities, including AF outcomes. Our aim was to compare the incidence of stroke and mortality by race and ethnicity among patients with incident AF managed in the VA. We hypothesized that individuals from minoritized racial and ethnic groups would have higher rates of stroke and mortality compared with White individuals.

## Methods

We conducted this study using the previously described REACH-AF (Race, Ethnicity, and Anticoagulant CHoice in Atrial Fibrillation) cohort.^21^ REACH-AF is a national retrospective cohort of patients enrolled in VA with incident, nonvalvular AF. This analysis included patients diagnosed from 2014 to 2021. The institutional review boards at the VA Pittsburgh Healthcare System and Greater Los Angeles VA approved the study. We followed the Strengthening the Reporting of Observational Studies in Epidemiology reporting guidelines.^22^

### Data source

We used administrative and clinical data from the VA Corporate Data Warehouse to identify the cohort and all relevant study variables. The Corporate Data Warehouse includes outpatient and inpatient clinical encounters, patient sociodemographic details, diagnosis codes, VA clinic stop codes (which are used to identify the clinical site of care), and all prescribed medications dispensed by VA pharmacies. We assessed patient clinical variables using inpatient and outpatient diagnosis codes within the 2 years before an index AF diagnosis.

### Cohort eligibility criteria

**Figure 1:**
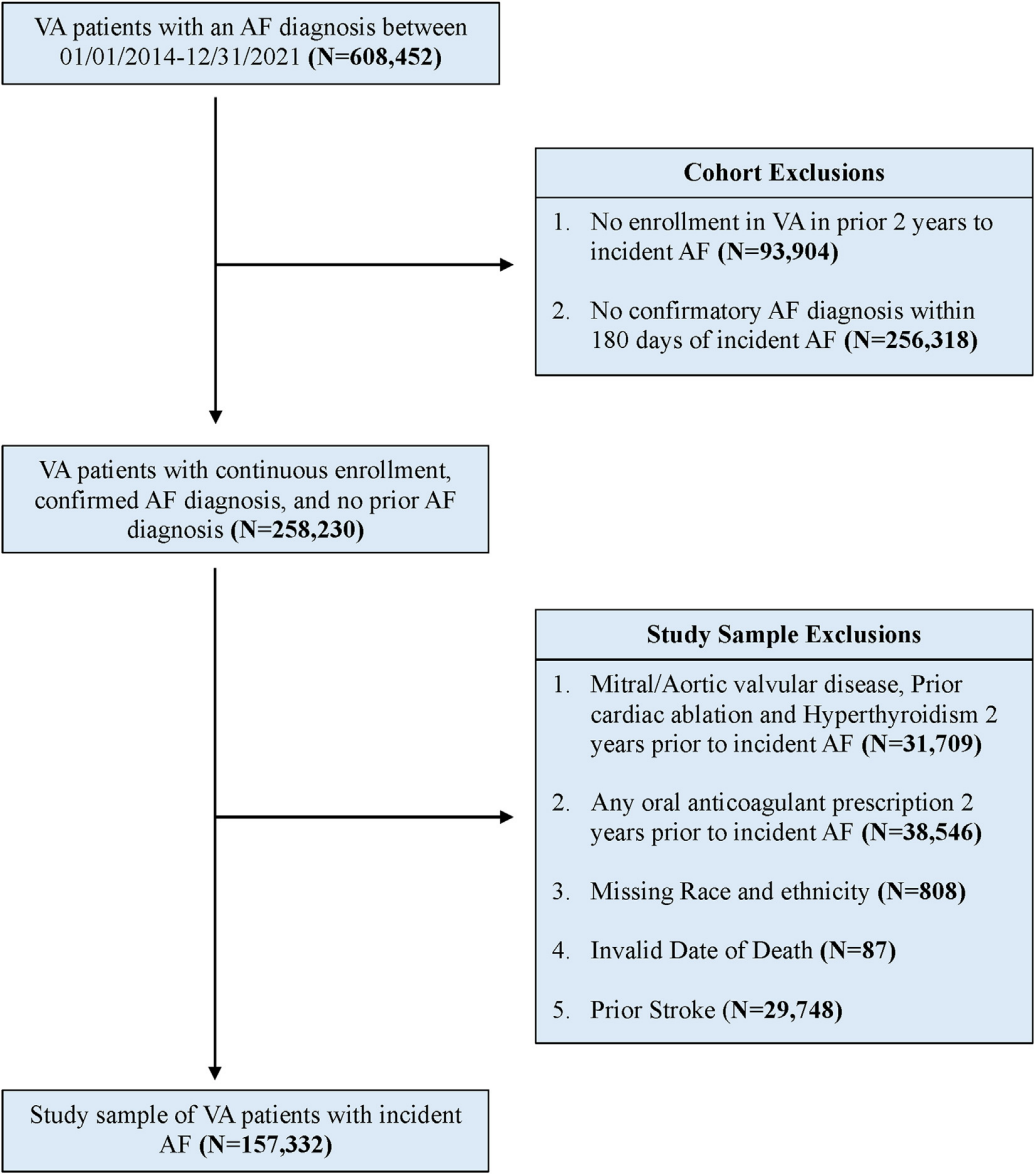
Identification of the Study Sample Among 608,452 patients in the VA with an index AF diagnosis from 2014 to 2021, we identified 258,230 patients with continuous VA enrollment, no prior enrollment in the VA in the prior 2 years to incident atrial fibrillation (AF), and no confirmatory AF diagnosis within 180 days after their index diagnosis. After applying additional exclusion criteria, the study cohort included 157,332 patients with incident AF. All exclusions were performed sequentially. AF = atrial fibrillation; VA = Veterans Health Administration.

To define the REACH-AF cohort, we used the established International Classification of Diseases, Ninth Revision and Tenth Revision diagnosis codes for AF from VA outpatient clinical encounters to include patients with an initial or index AF diagnosis from January 1, 2014, to December 31, 2021.^20^^,^^23^ We sequentially excluded patients who had any prior outpatient AF diagnosis or were not enrolled in VA for 2 years prior to their index diagnosis and those without 1 or more outpatient confirmatory diagnoses of AF in VA 7 to 180 days after their index diagnosis (Figure 1). To further define the study sample, we excluded patients with valvular AF using diagnosis codes for any form of aortic or mitral valvular disease, repair, or replacement 2 years prior to the index AF diagnosis.^21^ We also excluded patients with diagnoses of hyperthyroidism or prior stroke, cardiac ablation, or use of any anticoagulant therapy in the 2 years prior to AF diagnosis. Lastly, we excluded patients with missing race and ethnicity information (N = 808) and invalid dates of death.

### Study outcomes

Our 2 main study outcomes were ischemic stroke and all-cause mortality at any point within the study follow-up period (January 1, 2014, through May 31, 2022). The date of death was obtained using the VA Vital Status File, a combined mortality data file from the VA, Medicare, and Social Security.^24^, ^25^, ^26^ This combined dataset has a 98.3% sensitivity and 97.6% agreement with mortality dates from the National Death Index and captures deaths that occur outside VA through Social Security and Medicare.^27^ Stroke was defined using established International Classification of Diseases-9th Revision and -10th Revision diagnosis codes for ischemic stroke (Supplemental Table 1).^14^^,^^28^^,^^29^ For each patient, the date of the first occurrence of stroke or death after index AF diagnosis was identified. Follow-up time begins with the date of the initial AF diagnosis and ends with the event of death or stroke. For the stroke outcome, patients are also censored in the event of death. All patients who did not experience the event are censored at the end of the study period, May 31, 2022.

### Independent variables and covariates

We categorized our independent variables as race and ethnicity, including non-Hispanic American Indian/Alaska Native (AI/AN), non-Hispanic Asian, non-Hispanic Black, Hispanic, non-Hispanic Multiracial, or White, which served as the reference group using VA administrative data.^21^^,^^30^ Based on a health disparities research framework and previous AF disparities research,^21^^,^^31^ we examined baseline patient, clinician, and health care facility-level factors that may contribute to racial and ethnic disparities in health or health care and/or represent confounders of the associations between our independent and dependent variables of interest.

Patient sociodemographic factors included a categorical variable for age at index AF diagnosis, sex, and VA enrollment priority group (groups 1-8). The latter conveys veterans’ level of eligibility for VA services given their financial means, service-connected health conditions, and disabilities (lower groups have increased eligibility for VA benefits).^32^ We assessed geographic census region and level of rurality based on each individual’s residence. Given its association with treatment access and health outcomes, we assessed the Area Deprivation Index (ADI), a validated score comprising 17 socioeconomic indicators. We categorized the ADI into quintiles (1-5) using percentile rankings of all U.S. neighborhoods from least (1st percentile) to most (100th percentile) disadvantaged.^33^^,^^34^

We assessed patient clinical variables using inpatient and outpatient diagnosis codes within the 2 years before an index AF diagnosis. Using a modified Gagne comorbidity index, the comorbid conditions consisted of those associated with increased stroke risk and mortality including congestive heart failure, hypertension, diabetes, vascular disease, prior stroke, renal disease, liver disease, history of bleeding, hemiplegia, coagulopathy, chronic pulmonary disease, fluid and electrolyte disorders, pulmonary circulation disorders, HIV/AIDS, mental health, and substance use disorders.^30^^,^^35^ We also assessed frailty using a validated index defining individuals as nonfrail, prefrail, mildly, moderately, or severely frail based on conditions such as motor function difficulties, sensory loss, and cognitive impairment.^36^ We used the validated CHA_2_DS_2_-VASc prediction rule to categorize the 1-year stroke risk as low (score 0-1), moderate (score 2-4), and high (score >4) risk.^37^ We assessed variables in the HAS-BLED prediction rule for 1-year bleeding risk in AF that were not otherwise captured in the clinical variables, including use of medications predisposing to bleeding (ie, antiplatelet therapies such as aspirin and clopidogrel and nonsteroidal anti-inflammatory drugs).^38^ Because international normalized ratio levels were not available for the cohort, we do not report HAS-BLED scores; however, all other factors comprising this bleeding risk score are included in our model. We included a time-dependent variable for the use of anticoagulant therapy from the date of the index AF diagnosis until the date of the study outcome of interest or the end of the study period. We defined any anticoagulant therapy use as receipt of warfarin, apixaban, dabigatran, edoxaban, or rivaroxaban in VA outpatient settings. This variable was defined as having no gap in anticoagulant use from initiation of treatment to date of censoring >30 days. We used validated Current Procedural Terminology codes to account for catheter ablation and left atrial appendage occlusion procedures for AF management from the date of index AF diagnosis until the date of study outcome or the end of the study period. Lastly, for the mortality outcome, we included a time-dependent variable for an incident stroke, assessed from the date of index AF diagnosis until the date of death. For those who had incident stroke on the date of death, we assigned the date of stroke as 1 day earlier to allow for measurable time at risk of death following stroke.

Clinician- and facility-level variables included the outpatient clinical site where the index AF diagnosis was recorded (ie, primary care, cardiology, emergency department, and pharmacy) in addition to whether there was a clinical encounter with a cardiologist within 90 days of the index diagnosis.^29^ VA facility type was defined by where the clinician recorded an index AF diagnosis, including a VA medical center, primary care, multispecialty community-based outpatient clinic, and other. We also assessed the frequency of VA primary care visits in the year before an index AF diagnosis, categorized as <2 or ≥2.

### Statistical analysis

We compared baseline patient, clinician, and facility characteristics across race and ethnicity groups using chi-square tests for all categorical variables. We used Cox proportional hazard models to examine the relative hazard of stroke and mortality by race and ethnicity, with non-Hispanic White individuals as the reference group. We report incidence rates for each study outcome per 1,000 person-years. We created Kaplan-Meier plots of the 2 study outcomes by racial and ethnic groups and did not observe any substantial violation from the proportional hazards assumption. To adjust for facility-level differences in anticoagulant prescribing and/or VA formulary administration, all models incorporated a random effect for the VA clinical site where the index AF diagnosis was established.

Using a pharmacoequity research framework, we constructed models for each outcome using a 4-step process.^39^^,^^40^ This stepwise approach was used to gain an understanding of what level of factors most contribute to racial and ethnic variation in outcomes, starting with the least mutable factors to those most addressable. In step 1, we added patient demographic and clinical covariates (ie, medical comorbidities, body mass index, year of diagnosis, CHA_2_DS_2_-VASc stroke risk, bleeding risk, frailty, and use of AF procedures). For the mortality outcome, we also included a time-dependent stroke variable in this step. In step 2, we added a time-dependent anticoagulant use variable. In step 3, we added clinician and facility factors, including clinical site of index AF diagnosis, encounter with a cardiologist within 90 days, and frequency of VA primary care visits. Lastly, in step 4, we added patient socioeconomic covariates (ie, ADI and VA priority group) to the model. We conducted post hoc analyses to assess the effects of having an incident stroke on subsequent mortality, both overall and by race and ethnicity, by including interaction terms for incident stroke and race and ethnicity with mortality as the outcome in our model.

For all modeling steps, we determined the adjusted HRs (aHRs) and 95% CIs for racial and ethnic groups. For all analyses, we used a 2-tailed *P* < 0.05 to define statistical significance and conducted analyses using SAS Enterprise Guide 8.3 (SAS Institute).

## Results

### Baseline patient characteristics

Among 157,332 patients with incident AF in our cohort, the mean age was 72.9 ± 10.5 years, 97.8% were male, and racial and ethnic identities were 0.5% AI/AN, 1.2% Asian, 9.2% Black, 3.6% Hispanic, 0.6% multiracial, and 84.9% White (Table 1). The mean study follow-up period per patient was 3.1 years for stroke and 3.5 years for mortality. Black patients were younger and more likely to reside in the South; Hispanic and Black patients were more likely to live in disadvantaged neighborhoods and have greater rates of medical comorbidities, including high CHA_2_DS_2_VASc stroke risk scores (Table 1). All baseline patient demographic, clinical, clinician, and facility factors differed significantly at *P* < 0.001 across racial and ethnic groups, as highlighted in Table 1.

**Table 1:** Baseline Characteristics of Patients With Incident Atrial Fibrillation by Race and Ethnicity^a^

	Overall (N = 157,332)	AI/AN (n = 804)	Asian (n = 1,867)	Black (n = 14,427)	Hispanic (n = 5,709)	Multiracial (n = 876)	White (n = 133,649)
Sociodemographic characteristics							
Age at diagnosis (mean, SD)	72.9 (10.5)	70.3 (10.4)	71.8 (11.9)	68.0 (11.3)	71.8 (12.1)	70.2 (11.3)	73.5 (10.2)
18-64 y	27,374 (17.4)	182 (22.6)	425 (22.8)	5,256 (36.4)	1,312 (23.0)	225 (25.7)	19,974 (14.9)
65-74 y	66,280 (42.1)	378 (47.0)	745 (39.9)	5,538 (38.4)	2,111 (37.0)	365 (41.7)	57,143 (42.8)
75-84 y	40,491 (25.7)	174 (21.6)	386 (20.7)	2,397 (16.6)	1,387 (24.3)	197 (22.5)	35,950 (26.9)
≥85 y	23,187 (14.7)	70 (8.7)	311 (16.7)	1,236 (8.6)	899 (15.7)	89 (10.2)	20,582 (15.4)
Sex, n (%)							
Female	3,484 (2.2)	26 (3.2)	53 (2.8)	518 (3.6)	91 (1.6)	31 (3.5)	2,765 (2.1)
Male	153,848 (97.8)	778 (96.8)	1,814 (97.2)	13,909 (96.4)	5,618 (98.4)	845 (96.5)	130,884 (97.9)
Region of residence							
Midwest	39,042 (24.8)	152 (18.9)	222 (11.9)	2,653 (18.4)	358 (6.3)	158 (18.0)	35,499 (26.6)
Northeast	23,530 (15.0)	78 (9.7)	103 (5.5)	1,580 (11.0)	454 (8.0)	144 (16.4)	21,171 (15.8)
South	62,442 (39.7)	292 (36.3)	488 (26.1)	8,485 (58.8)	1,818 (31.8)	385 (43.9)	50,974 (38.1)
West	29,367 (18.7)	269 (33.5)	971 (52.0)	1,594 (11.0)	1,740 (30.5)	181 (20.7)	24,612 (18.4)
Outside the 50 states and DC	1,019 (0.6)	–	12 (0.6)	15 (0.1)	899 (15.7)	–	91 (0.1)
Rurality, n (%)							
Large metro	64,048 (40.7)	264 (32.8)	922 (49.4)	8,729 (60.5)	3,189 (55.9)	343 (39.2)	50,601 (37.9)
Metropolitan	19,654 (12.5)	128 (15.9)	162 (8.7)	808 (5.6)	266 (4.7)	119 (13.6)	18,171 (13.6)
Small metro	56,094 (35.7)	290 (36.1)	617 (33.0)	4,190 (29.0)	1,665 (29.2)	341 (38.9)	48,991 (36.7)
Noncore rural	15,577 (9.9)	110 (13.7)	89 (4.8)	594 (4.1)	147 (2.6)	66 (7.5)	14,571 (10.9)
Area Deprivation Index (mean, SD)	55.5 (25.5)	58.8 (25.8)	38.7 (28.1)	62.9 (27.0)	57.5 (28.2)	56.8 (26.2)	54.8 (25.0)
Quintile 1 (1-29)	28,691 (18.2)	135 (16.8)	838 (44.9)	2,060 (14.3)	1,160 (20.3)	158 (18.0)	24,340 (18.2)
Quintile 2 (30-46)	30,265 (19.2)	120 (14.9)	318 (17.0)	2,153 (14.9)	892 (15.6)	165 (18.8)	26,617 (19.9)
Quintile 3 (47-62)	31,090 (19.8)	155 (19.3)	234 (12.5)	2,343 (16.2)	882 (15.4)	149 (17.0)	27,327 (20.4)
Quintile 4 (63-78)	30,804 (19.6)	162 (20.1)	195 (10.4)	2,544 (17.6)	879 (15.4)	182 (20.8)	26,842 (20.1)
Quintile 5 (79-100)	35,528 (22.6)	226 (28.1)	236 (12.6)	5,220 (36.2)	1,721 (30.1)	213 (24.3)	27,912 (20.9)
VA enrollment priority group							
Group 1-3	73,512 (46.7)	429 (53.4)	1,126 (60.3)	7,667 (53.1)	2,817 (49.3)	464 (53.0)	61,009 (45.6)
Group 4	3,122 (2.0)	16 (2.0)	31 (1.7)	355 (2.5)	194 (3.4)	15 (1.7)	2,511 (1.9)
Group 5	37,151 (23.6)	192 (23.9)	321 (17.2)	3,796 (26.3)	1,602 (28.1)	215 (24.5)	31,025 (23.2)
Group 6	5,642 (3.6)	24 (3.0)	44 (2.4)	248 (1.7)	108 (1.9)	21 (2.4)	5,197 (3.9)
Group 7-8	33,487 (21.3)	116 (14.4)	308 (16.5)	1,886 (13.1)	717 (12.6)	140 (16.0)	30,320 (22.7)
Clinical characteristics							
Selected medical comorbidities							
Congestive heart failure	23,464 (14.9)	126 (15.7)	295 (15.8)	3,446 (23.9)	956 (16.7)	152 (17.4)	18,489 (13.8)
Hypertension	114,673 (72.9)	571 (71.0)	1,314 (70.4)	11,619 (80.5)	4,327 (75.8)	668 (76.3)	96,174 (72.0)
Diabetes	56,721 (36.1)	338 (42.0)	768 (41.1)	6,188 (42.9)	2,505 (43.9)	333 (38.0)	46,589 (34.9)
Vascular disease	45,449 (28.9)	241 (30.0)	479 (25.7)	3,968 (27.5)	1,618 (28.3)	251 (28.7)	38,892 (29.1)
History of bleeding	31,765 (20.2)	148 (18.4)	350 (18.7)	3,327 (23.1)	1,315 (23.0)	204 (23.3)	26,421 (19.8)
Liver disease	8,168 (5.2)	49 (6.1)	107 (5.7)	1,039 (7.2)	422 (7.4)	53 (6.1)	6,498 (4.9)
Kidney disease	15,986 (10.2)	89 (11.1)	206 (11.0)	2,486 (17.2)	696 (12.2)	119 (13.6)	12,390 (9.3)
Mental health disorder	46,238 (29.4)	276 (34.3)	533 (28.5)	5,185 (35.9)	2,103 (36.8)	332 (37.9)	37,809 (28.3)
Substance use disorder	22,356 (14.2)	137 (17.0)	191 (10.2)	3,167 (22.0)	803 (14.1)	147 (16.8)	17,911 (13.4)
Hemiplegia	1,143 (0.7)	–	15 (0.8)	167 (1.2)	53 (0.9)	–	900 (0.7)
Coagulopathy	5,268 (3.3)	22 (2.7)	49 (2.6)	541 (3.7)	265 (4.6)	30 (3.4)	4,361 (3.3)
Chronic pulmonary disease	39,904 (25.4)	232 (28.9)	356 (19.1)	3,565 (24.7)	1,175 (20.6)	236 (26.9)	34,340 (25.7)
Fluid and electrolyte disorders	19,523 (12.4)	109 (13.6)	226 (12.1)	2,618 (18.1)	886 (15.5)	141 (16.1)	15,543 (11.6)
Pulmonary circulation disorders	3,721 (2.4)	16 (2.0)	42 (2.2)	628 (4.4)	151 (2.6)	20 (2.3)	2,864 (2.1)
HIV/AIDS	429 (0.3)	–	–	171 (1.2)	25 (0.4)	–	225 (0.2)
Oral anticoagulant use	120,114 (76.3)	597 (74.3)	1,296 (69.4)	10,590 (73.4)	4,180 (73.2)	665 (75.9)	102,786 (76.9)
Warfarin	24,829 (20.7)	135 (22.6)	247 (19.1)	2,485 (23.5)	1,033 (24.7)	139 (20.9)	20,790 (20.2)
Direct oral anticoagulant	95,285 (79.3)	462 (77.4)	1,049 (80.9)	8,105 (76.5)	3,147 (75.3)	526 (79.1)	81,996 (79.8)
Medications predisposing to bleeding^b^	71,914 (45.7)	390 (48.5)	888 (47.6)	8,753 (60.7)	3,262 (57.1)	457 (52.2)	58,164 (43.5)
BMI (mean, SD)	30.5 (6.8)	31.4 (6.5)	29.4 (6.5)	30.7 (7.6)	30.1 (6.6)	31.1 (7.3)	30.5 (6.7)
<18.5 kg/m^2^	1,730 (1.1)	10 (1.2)	30 (1.6)	304 (2.1)	80 (1.4)	13 (1.5)	1,293 (1.0)
18.5-<25 kg/m^2^	28,718 (18.3)	95 (11.8)	427 (22.9)	2,900 (20.1)	1,119 (19.6)	139 (15.9)	24,038 (18.0)
25-<30 kg/m^2^	51,358 (32.6)	257 (32.0)	670 (35.9)	4,143 (28.7)	1,943 (34.0)	261 (29.8)	44,084 (33.0)
30-<35 kg/m^2^	39,802 (25.3)	234 (29.1)	415 (22.2)	3,476 (24.1)	1,416 (24.8)	237 (27.1)	34,024 (25.5)
35-<40 kg/m^2^	20,046 (12.7)	114 (14.2)	182 (9.7)	1,923 (13.3)	641 (11.2)	113 (12.9)	17,073 (12.8)
≥40 kg/m^2^	12,802 (8.1)	80 (10.0)	111 (5.9)	1,439 (10.0)	450 (7.9)	96 (11.0)	10,626 (8.0)
CHA2DS2-VASc stroke risk score (mean, SD)	3 (1.4)	3 (1.4)	3 (1.4)	3 (1.5)	3 (1.5)	3 (1.3)	3 (1.3)
Median (IQR)	3 (2-4)	3 (2-4)	3 (2-4)	3 (2-4)	3 (2-4)	3 (2-4)	3 (2-4)
0-1, n (%)	27,190 (17.3)	163 (20.3)	364 (19.5)	3,130 (21.7)	1,053 (18.4)	174 (19.9)	22,306 (16.7)
2-4, n (%)	113,499 (72.1)	552 (68.7)	1,301 (69.7)	9,698 (67.2)	3,903 (68.4)	624 (71.2)	97,421 (72.9)
>4, n (%)	16,643 (10.6)	89 (11.1)	202 (10.8)	1,599 (11.1)	753 (13.2)	78 (8.9)	13,922 (10.4)
Year of AF diagnosis							
2014	16,939 (10.8)	90 (11.2)	182 (9.7)	1,514 (10.5)	578 (10.1)	87 (9.9)	14,488 (10.8)
2015	18,547 (11.8)	81 (10.1)	223 (11.9)	1,608 (11.1)	719 (12.6)	96 (11.0)	15,820 (11.8)
2016	20,112 (12.8)	106 (13.2)	234 (12.5)	1,782 (12.4)	723 (12.7)	107 (12.2)	17,160 (12.8)
2017	21,989 (14.0)	109 (13.6)	236 (12.6)	1,932 (13.4)	790 (13.8)	136 (15.5)	18,786 (14.1)
2018	23,268 (14.8)	120 (14.9)	277 (14.8)	2,134 (14.8)	822 (14.4)	137 (15.6)	19,778 (14.8)
2019	22,721 (14.4)	121 (15.0)	278 (14.9)	2,100 (14.6)	827 (14.5)	126 (14.4)	19,269 (14.4)
2020	15,268 (9.7)	77 (9.6)	183 (9.8)	1,534 (10.6)	495 (8.7)	76 (8.7)	12,903 (9.7)
2021	18,488 (11.8)	100 (12.4)	254 (13.6)	1,823 (12.6)	755 (13.2)	111 (12.7)	15,445 (11.6)
Frailty index classification							
Nonfrail	62,971 (40.0)	301 (37.4)	783 (41.9)	4,912 (34.0)	2,003 (35.1)	299 (34.1)	54,673 (40.9)
Prefrail	54,219 (34.5)	265 (33.0)	615 (32.9)	4,923 (34.1)	1,935 (33.9)	339 (38.7)	46,142 (34.5)
Mildly frail	26,292 (16.7)	150 (18.7)	319 (17.1)	2,842 (19.7)	1,132 (19.8)	161 (18.4)	21,688 (16.2)
Moderately frail	9,711 (6.2)	64 (8.0)	108 (5.8)	1,202 (8.3)	433 (7.6)	60 (6.8)	7,844 (5.9)
Severely frail	4,139 (2.6)	24 (3.0)	42 (2.2)	548 (3.8)	206 (3.6)	17 (1.9)	3,302 (2.5)
Atrial fibrillation procedures^c^	5,428 (3.5)	27 (3.4)	58 (3.1)	585 (4.1)	193 (3.4)	34 (3.9)	4,531 (3.4)
Clinician and facility characteristics							
Specialty of initial AF diagnosis provider							
Cardiology	19,555 (12.4)	94 (11.7)	256 (13.7)	2,348 (16.3)	1,012 (17.7)	141 (16.1)	15,704 (11.8)
Emergency department	21,895 (13.9)	118 (14.7)	211 (11.3)	2,731 (18.9)	1,063 (18.6)	122 (13.9)	17,650 (13.2)
Primary care	80,634 (51.3)	412 (51.2)	1,033 (55.3)	5,939 (41.2)	2,572 (45.1)	427 (48.7)	70,251 (52.6)
Pharmacy	21,930 (13.9)	105 (13.1)	190 (10.2)	2,000 (13.9)	585 (10.2)	104 (11.9)	18,946 (14.2)
Other	13,318 (8.5)	75 (9.3)	177 (9.5)	1,409 (9.8)	477 (8.4)	82 (9.4)	11,098 (8.3)
Facility type							
VA medical center	102,642 (65.2)	524 (65.2)	1,223 (65.5)	11,149 (77.3)	4,081 (71.5)	585 (66.8)	85,080 (63.7)
Primary care CBOC	16,704 (10.6)	87 (10.8)	265 (14.2)	899 (6.2)	398 (7.0)	78 (8.9)	14,977 (11.2)
Multispecialty CBOC	28,636 (18.2)	129 (16.0)	293 (15.7)	1,777 (12.3)	949 (16.6)	165 (18.8)	25,323 (18.9)
Other	8,992 (5.7)	60 (7.5)	84 (4.5)	578 (4.0)	272 (4.8)	45 (5.1)	7,953 (6.0)
Cardiology visit ≤90 d of AF	77,041 (49.0)	420 (52.2)	899 (48.2)	8,439 (58.5)	3,427 (60.0)	460 (52.5)	63,396 (47.4)
≥2 Primary care visits within a year	117,357 (74.6)	615 (76.5)	1,347 (72.1)	11,522 (79.9)	4,597 (80.5)	684 (78.1)	98,592 (73.8)

- = sample sizes <10 that are too small to report.

AF = atrial fibrillation; AI/AN = American Indian/Alaska Native; BMI = body mass index; CBOC = community-based outpatient clinic; DC = District of Columbia; VA = Veterans Health Administration.

a All baseline characteristics differed significantly (*P* < 0.001) across racial and ethnic groups.

b Medications predisposing to bleeding refer to antiplatelet therapies and nonsteroidal anti-inflammatory drugs.

c Atrial fibrillation procedures include catheter ablation and left atrial appendage occlusion.

### Incidence of stroke by race and ethnicity

**Figure 2:**
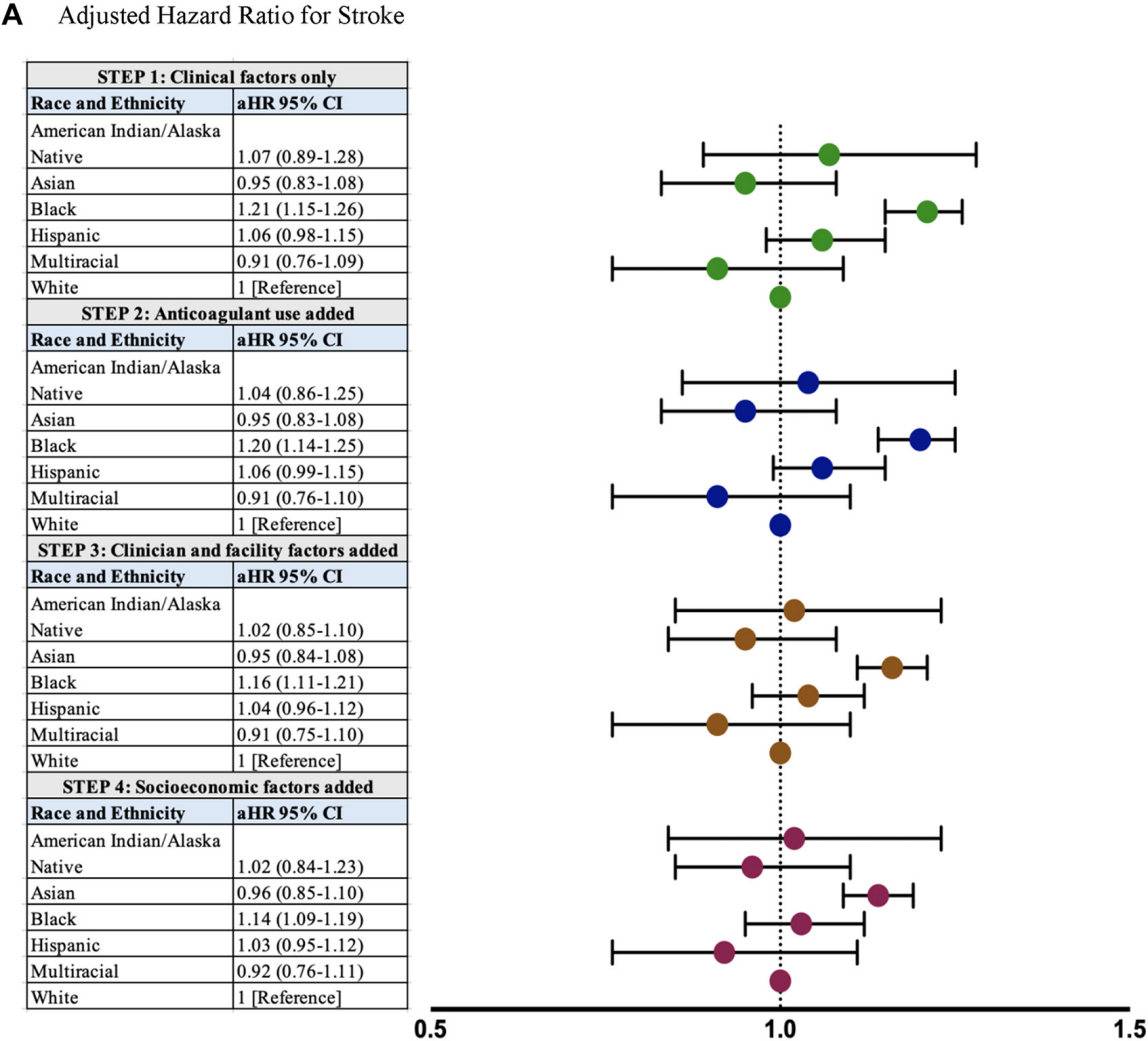

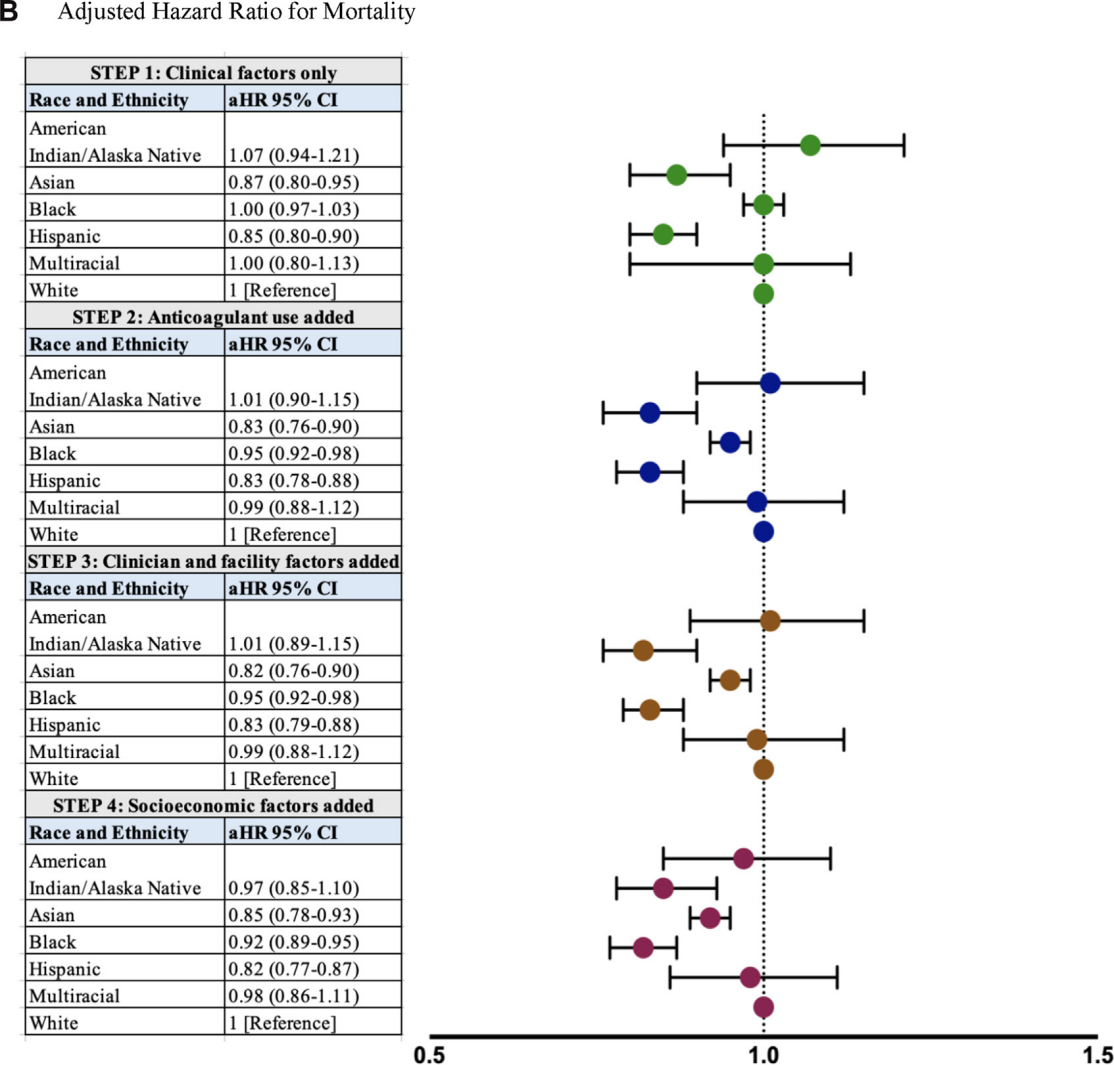
Association of Race and Ethnicity With Stroke and Mortality The adjusted rates of (A) stroke and (B) death by race and ethnicity are displayed. Using the Cox proportional hazards modeling approach, considering patient demographic and clinical factors only (step 1), adding anticoagulant use (step 2), clinician and facility factors (step 3), and patient socioeconomic factors (step 4), Black patients had significantly higher aHRs of stroke than White patients (A); Asian, Black, and Hispanic patients had lower aHRs for mortality than White patients (B). For stroke and mortality outcomes, there were no statistically significant differences observed between American Indian/Alaska Native and multiracial patients with White patients as their comparator. aHR = adjusted HR.

Overall, 22,628 (14.7%) individuals developed a stroke during the study period, reflecting a stroke incidence rate of 46.3 per 1,000 person-years of follow-up in the cohort. Stroke incidence rates varied significantly by race and ethnicity (*P* < 0.001), with the highest rates observed in Black (55.7), Hispanic (52.4), and AI/AN (47.3) individuals (Table 2). In step 1 of our model, adjusting for patient demographic and clinical factors, Black individuals (aHR: 1.21; 95% CI: 1.15-1.26) were significantly more likely than White individuals to develop stroke (Figure 2A). After further adjusting for anticoagulant use (step 2), clinician and facility factors (step 3), and socioeconomic factors (step 4), Black individuals continued to have a significantly higher incidence of stroke (aHR: 1.14; 95% CI: 1.09-1.20). Across all modeling steps, there were no statistically significant differences in stroke observed between White and AI/AN, Asian, Hispanic, or multiracial individuals (Figure 2A).

**Table 2:** Incidence of Stroke and Mortality by Race and Ethnicity

	Stroke			Mortality		
	Number of Events	Person-Time, y	Rate (per 1,000 Person-Years)	Number of Events	Person-Time, y	Rate (per 1,000 Person-Years)
American Indian/Alaska Native	3	2,535.4	47.3	261	2,779.9	93.4
Asian	251	5,840.1	43.0	564	6,496.3	86.8
Black	2,418	43,439.3	55.7	4,492	49,111.3	91.5
Hispanic	929	17,742.7	52.3	1,707	20,009.0	85.3
Multiracial	114	2,808.9	40.6	276	3,033.0	91.0
White	18,796	416,729.1	45.1	44,988	462,658.3	97.2
Overall	22,626	489,095.5	46.3	52,288	544,087.8	96.1

### Incidence of mortality by race and ethnicity

Overall, 52,288 (33.2%) individuals died during the study period, reflecting a mortality incidence rate of 96.1 per 1,000 person-years of follow-up in the cohort. Mortality incidence rates varied significantly by race and ethnicity (*P* < 0.001), with the highest rates observed in White individuals (97.3), followed by AI/AN (93.4), Black (91.5), Asian (86.8), and Hispanic (85.3) individuals (Table 2). In step 1 of our model, adjusting for patient demographic and clinical factors, Black and White individuals had similar mortality risk (aHR: 1.00; 95% CI: 0.97-1.03) (Figure 2B). After further adjusting for anticoagulant use (step 2), Black individuals had lower mortality risk than White individuals (aHR: 0.95; 95% CI: 0.92-0.98), a finding that persisted with further adjusting for clinician, facility, and socioeconomic factors (Figure 2B). Across all adjusted models, including in the final fully adjusted model, Asian individuals (aHR: 0.85; 95% CI: 0.78-0.93) and Hispanic individuals (aHR: 0.82; 95% CI: 0.77-0.87) had significantly lower risk of death than White individuals (Figure 2B). We did not observe statistically significant mortality differences between White and AI/AN or multiracial individuals across any of the modeling steps (Figure 2B).

In fully adjusted models, incident stroke was independently associated with mortality (aHR: 1.41; 95% CI: 1.37-1.46). In post hoc analyses, the interaction between race and ethnicity and stroke was significantly associated with mortality. Among those with stroke, adjusted mortality rates were higher for all racial and ethnic minoritized groups; the interactions were statistically significant for all but the Asian group. In contrast, among those without stroke, mortality rates were lower for all racial and ethnic minoritized groups; the interactions were statistically significant for Black, Hispanic, and Asian individuals (Supplemental Table 2).

## Discussion

In a national cohort of 157,332 patients with incident AF managed in the VA between 2014 and 2022, we observed significant racial and ethnic differences in the incidence of stroke and mortality (Central Illustration). First, we found that Black individuals had a 14% higher adjusted risk of stroke than White individuals. Second, the magnitude of the association narrowed across modeling steps for our stroke outcome, suggesting that provider, facility, and socioeconomic factors beyond traditionally assessed clinical risk factors may be associated with serious outcomes for AF. Third, we found that Asian, Black, and Hispanic individuals had 8% to 18% lower adjusted risks of death than White individuals in fully adjusted models. Fourth, we found that mortality risk decreased for Black compared with White patients when adjusting for anticoagulant use, suggesting a potential disparity-lowering effect of equitable therapy use.

**Central Illustration:**
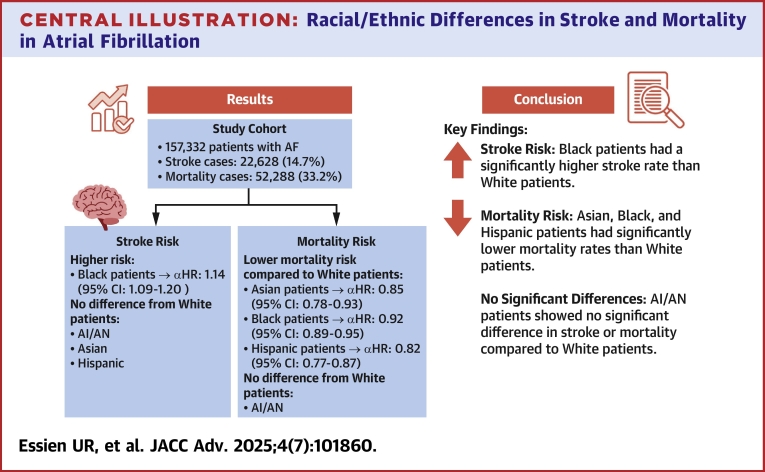
Racial/Ethnic Differences in Stroke and Mortality in Atrial Fibrillation The illustration portrays the key findings within this study's cohort on stroke and mortality risk. Black patients had significantly higher stroke rates, while Asian, Black, and Hispanic patients had significantly lower mortality rates than White patients. AF = atrial fibrillation; aHR = adjusted HR; AI/AN = American Indian/Alaska Native.

Using contemporary data from the largest integrated health system in the United States, our analysis extends prior work examining racial disparities in AF-related outcomes. In data from the national U.S. Centers for Medicare & Medicaid Services from 2010 to 2011, researchers observed higher rates of stroke in Black patients than White patients with AF.^28^ They also observed lower rates of mortality in Hispanic patients with no difference between Black and White patients. These prior findings, generated over a decade before the broad diffusion of several newer guideline-recommended therapies for stroke prevention in AF, closely mirror our findings and identify an urgent need to equitably implement evidence-based therapies using established quality improvement models for AF. One such quality improvement model has been the Get With the Guidelines-AFib Registry, which collects data nationally on hospitalized patients with AF.^41^ Yet, in a recent analysis of this registry, Black patients also experienced significantly higher rates of stroke with slightly higher rates of mortality outcomes than White patients.^13^ Notably, these mortality differences did not persist for patients receiving anticoagulant therapy. In a smaller, regional sample of 15,080 study participants of the ARIC (Atherosclerosis Risk In Communities) study,^17^ the authors found a nearly 2-fold higher rate of stroke and a 1.5-fold higher risk of mortality in Black compared to White individuals with AF.^17^ However, in adjusted models, the mortality difference by race observed was minimal. Notably, this analysis did not control for anticoagulant use or the breadth of social factors captured in the present analysis.

Our findings of higher rates of stroke in Black patients, even when controlling for anticoagulant use, suggest that there are other mechanisms by which stroke prevention must be addressed in ongoing VA quality improvement efforts for AF. These include improving equitable access to all AF treatment strategies beyond anticoagulation, including rhythm control agents and therapeutic procedures such as cardiac ablation, for which notable disparities have been observed.^42^^,^^43^ Extant research has reported poor risk factor control among racially and ethnically minoritized groups, and implementing strategies to support risk factor management, including through traditionally anticoagulation-focused pharmacy clinics, may help improve stroke outcome disparities.^44^ Furthermore, our observation that the magnitude of the association between race and stroke decreased with the addition of socioeconomic markers in our stepwise modeling suggests that addressing social factors and structural determinants beyond the health system may improve downstream AF outcomes. Such factors, including the impact of neighborhood deprivation, income level, and disability, have been described in past AF investigations and represent potential intervention targets to eliminate stroke disparities in AF.^45^, ^46^, ^47^ These determinants also further include negative physician biases toward minoritized individuals, which are not only associated with treatment-related outcomes but also patient health outcomes.^48^^,^^49^

Our finding that racial and ethnic minoritized patients had lower rates of mortality than White patients in our cohort is noteworthy, particularly in this cohort of older adults with AF.^50^ Notably, within VA, prior work has demonstrated lower mortality for Black patients with chronic conditions such as heart failure and more acute conditions including pneumonia.^28^^,^^51^, ^52^, ^53^ This “VA mortality paradox” may reflect improved access to overall and specialist care in VA compared to non-VA, lower cost of care improving overall access to pharmacotherapies, as well as improved wrap-around services and focus on the social and structural determinants of health. Nevertheless, the data on AF disparities in mortality are mixed and warrant further study.^17^^,^^28^^,^^51^^,^^52^^,^^54^^,^^55^ AF is a disease of the elderly, and prior work has suggested wide variation in the lifespan of minoritized adults. One hypothesis may be that minoritized adults who live long enough may experience a higher life expectancy benefit than those who are younger, thus reversing traditionally reported mortality trends.^56^ Identifying specific strategies that have helped to improve AF mortality outcomes for historically underserved racial and ethnic groups with AF will be critical for future research. Furthermore, our observation that Black and Hispanic individuals with stroke had a significantly higher mortality rate, whereas among those without stroke, Asian, Black, and Hispanic individuals had a significantly lower risk for this outcome, suggests that stroke is moderating the relationship between race, ethnicity, and mortality. These findings emphasize the importance of equitably implementing early, effective stroke prevention strategies for patients with AF, particularly those from minoritized backgrounds with excess mortality.

### Study Limitations

There are limitations to consider for this study. First, we examined predominantly male patients receiving medical care within the VA, which limits the generalizability of our findings to non-VA health care settings or practices responsible for managing larger proportions of female patients with AF. Second, data used to identify AF diagnoses and our primary stroke outcomes were solely through the VA and did not include non-VA data. Third, these data did not include specific cause of death, which precluded analyses of cardiovascular mortality. Fourth, the analysis did not consider treatment factors outside of the VA health system from Medicare, Medicaid, or commercial insurers, such as the ascertainment of anticoagulant medication use by patients surrounding their outcomes, which may bias our results.^54^ Furthermore, we did not assess inpatient anticoagulant use, nor did we assess anticoagulation adherence. Fifth, the limited sample of AI/AN and multiracial individuals may have precluded us from identifying statistically significant differences in stroke or mortality outcomes compared with White patients. Nonetheless, this is one of the few analyses that have included these racial groups in the assessment of AF outcomes.^14^

## Conclusions

In a national cohort of patients with incident AF managed in the VA, Black patients had significantly higher rates of stroke than White patients. We also found that Asian, Black, and Hispanic patients had significantly lower mortality rates compared with White patients. Understanding the determinants of these racial and ethnic differences in health outcomes is essential to improving the health and health care for an increasingly diverse patient population with AF managed in the largest U.S. integrated health care system.

Perspectives **COMPETENCY IN SYSTEMS-BASED PRACTICE:** AF is the most common heart rhythm disorder. Despite improved pharmacotherapies and procedures to manage this condition, there remain racial and ethnic disparities in associated AF outcomes, including in stroke and mortality. It is imperative for clinicians to be aware of potential biases that can be associated with variation in treatment practices and address disparities to improve care for all patients with AF. Our work ties into the following clinical competencies: medical knowledge, patient care, and systems based Practice.

**TRANSLATIONAL OUTLOOK:** Even within a uniform health system that largely eliminates the burden of the cost of care, health disparities persist for patients with AF. Improving AF treatment disparities is a significant, long-term process in which clinicians and researchers will have a substantial part. Effective implementation of novel interventions is needed to ensure equitable management and outcomes for the increasing number of patients with AF.

## Funding support and author disclosures

The funding was from the Veterans Affairs Health Services Research and Development Division (CDA-20-049) and the 10.13039/100000968American Heart Association (Amos Medical Faculty Development Program Award). The funder had no role in the design and conduct of the study; collection, management, analysis, and interpretation of the data; preparation, review, or approval of the manuscript; and decision to submit the manuscript for publication. The authors have reported that they have no relationships relevant to the contents of this paper to disclose.
