# Supplementary material for: Association of Race and Ethnicity With Stroke and Mortality Outcomes in Atrial Fibrillation
Source: JACC Adv. 2025 Jun 5;4(7):101860. doi: 10.1016/j.jacadv.2025.101860 (PMC12173103; doi:10.1016/j.jacadv.2025.101860)
Supplement: Supplemental Material [file mmc1.docx]

**Supplemental Table 1. ICD Codes for Ischemic Stroke**

| **ICD-9-CM** | **ICD-10-CM** |
| --- | --- |
| \| 433.0 \| Occlusion and stenosis of basilar artery \| \| --- \| --- \| \| 433.00 \| Occlusion & stenosis of basilar artery, w/o mention of cerebral infarction \| \| 433.01 \| Occlusion & stenosis of basilar artery, w/cerebral infarction \| \| 433.1 \| Occlusion and stenosis of carotid artery \| \| 433.10 \| Occlusion & stenosis of carotid artery, w/o cerebral infarction \| \| 433.11 \| Occlusion & stenosis of carotid artery, w/ cerebral infarction \| \| 433.2 \| Occlusion and stenosis of vertebral artery \| \| 433.20 \| Occlusion & stenosis of vertebral artery, w/o mention of cerebral infarction \| \| 433.21 \| Occlusion & stenosis of vertebral artery, w/ cerebral infarction \| \| 433.3 \| Occlusion and stenosis of multiple and bilateral precerebral arteries \| \| 433.30 \| Occlusion & stenosis fo multiple & bilateral arteries, w/o mention of cerebral infarction \| \| 433.31 \| Occlusion& stenosis of multiple & bilateral arteries, w/ cerebral infarction \| \| 433.8 \| Occlusion and stenosis of other specified precerebral artery \| \| 433.80 \| Occlusion & stenosis of other specified precerebral artery, w/o mention of cerebral infarction \| \| 433.81 \| Occlusion & stenosis of other specified precerebral artery, w/ cerebral infarction \| \| 433.9 \| Occlusion and stenosis of unspecified precerebral artery \| \| 433.90 \| Occlusion & stenosis of unspecified precerebral artery w/o mention of cerebral infarction \| \| 433.91 \| Occlusion & stenosis of unspecified precerebral artery w/ cerebral infarction \| \| 434.0 \| Cerebral thrombosis \| \| 434.00 \| Cerebral thrombosis w/o mention of cerebral infarction \| \| 434.01 \| Cerebral thrombosis w/ cerebral infarction \| \| 434.1 \| Cerebral embolism \| \| 434.10 \| Cerebral embolism w/o mention of cerebral infarction \| \| 434.11 \| Cerebral embolism w/ cerebral infarction \| \| 434.9 \| Cerebral artery occlusion, unspecified \| \| 434.90 \| Cerebral artery occlusion, unspecified, w/o mention of cerebral infarction \| \| 434.91 \| Cerebral artery occlusion, unspecified, w/ cerebral infarction \| \| 435.0 \| Basilar artery syndrome \| \| 435.1 \| Vertebral artery syndrome \| \| 435.2 \| Subclavian steal syndrome \| \| 435.3 \| Vertebrobasilar artery syndrome \| \| 435.8 \| Other specified transient cerebral ischemias \| \| 435.9 \| Unspecified transient cerebral ischemia \| \| 436. \| Acute, but ill-defined, cerebrovascular disease \| \| 437.0 \| Cerebral atherosclerosis \| \| 437.1 \| Other generalized ischemic cerebrovascular disease \| \| 437.2 \| Hypertensive encephalopathy \| \| 437.3 \| Cerebral aneurysm, nonruptured \| \| 437.4 \| Cerebral arteritis \| \| 437.5 \| Moyamoya disease \| \| 437.6 \| Nonpyogenic thrombosis of intracranial venous sinus \| \| 437.7 \| Transient global amnesia \| \| 437.8 \| Other ill-defined cerebrovascular disease \| \| 437.80 \| Cerebral/brain quadriplegia \| \| 437.81 \| Cerebral/brain paraplegia \| \| 437.89 \| Other cerebrovascular disease, nec \| \| 437.9 \| Unspecified cerebrovascular disease \| \| 438. \| Late effects of cerebrovascular disease \| \| 438.0 \| Late effect of cerebrovascular disease, cognitive deficits \| \| 438.10 \| Late effect of cerebrovascular disease, speech and language deficits, unspecified \| \| 438.11 \| Late effect of cerebrovascular disease, speech and language deficits, aphasia \| \| 438.12 \| Late effect of cerebrovascular disease, speech and language deficits, dysphasia \| \| 438.13 \| Late effects of cerebrovascular disease, dysarthria \| \| 438.14 \| Late effects of cerebrovascular disease, fluency disorder \| \| 438.19 \| Late effect of cerebrovascular disease, other speech and language deficits \| \| 438.20 \| Late effect of cerebrovascular disease, hemiplegia affecting uspecified side \| \| 438.21 \| Late effect of cerebrovascular disease, hemiplegia affecting dominant side \| \| 438.22 \| Late effect of cerebrovascular disease, hemiplegia affecting nondominant side \| \| 438.30 \| Late effect of cerebrovascular disease, monoplegia of upper limb affecting unspecified side \| \| 438.31 \| Late effect of cerebrovascular disease, monoplegia of upper limb affecting dominant side \| \| 438.32 \| Late effect of cerebrovascular disease, monoplegia of upper limb affecting nondominant side \| \| 438.40 \| Late effect of cerebrovascular disease, monoplegia of lower limb affecting unspecified side \| \| 438.41 \| Late effect of cerebrovascular disease, monoplegia of lower limb affecting dominant side \| \| 438.42 \| Late effect of cerebrovascular disease, monoplegia of lower limb affecting nondominant side \| \| 438.50 \| Late effect of cerebrovascular disease, other paralytic syndrome affecting unspecified side \| \| 438.51 \| Late effect of cerebrovascular disease, other paralytic syndrome affecting dominant side \| \| 438.52 \| Late effect of cerebrovascular disease, other paralytic syndrome affecting nondominant side \| \| 438.53 \| Other paralytic syndrome, bilateral \| \| 438.6 \| Alterations of sensations \| \| 438.7 \| Disturbances of vision \| \| 438.81 \| Other late effect of cerebrovascular disease, apraxia \| \| 438.82 \| Other late effect of cerebrovascular disease, dysphagia \| \| 438.83 \| Facial weakness \| \| 438.84 \| Ataxia \| \| 438.85 \| Vertigo \| \| 438.89 \| Other late effects of cerebrovascular disease \| \| 438.9 \| Unspecified late effects of cerebrovascular disease \| \| V12.54 \| Personal history of transient ischemic attack (tia), and cerebral infarction without residual deficits \| \|  \|  \| \|  \|  \| \|  \|  \| \|  \|  \| \|  \|  \| \|  \|  \| | \| I63.00 \| Cerebral infarction due to thrombosis of unspecified precerebral artery \| \| --- \| --- \| \| I63.011 \| Cerebral infarction due to thrombosis of right vertebral artery \| \| I63.012 \| Cerebral infarction due to thrombosis of left vertebral artery \| \| I63.013 \| Cerebral infarction due to thrombosis of bilateral vertebral arteries \| \| I63.019 \| Cerebral infarction due to thrombosis of unspecified vertebral artery \| \| I63.02 \| Cerebral infarction due to thrombosis of basilar artery \| \| I63.031 \| Cerebral infarction due to thrombosis of right carotid artery \| \| I63.032 \| Cerebral infarction due to thrombosis of left carotid artery \| \| I63.033 \| Cerebral infarction due to thrombosis of bilateral carotid arteries \| \| I63.039 \| Cerebral infarction due to thrombosis of unspecified carotid artery \| \| I63.09 \| Cerebral infarction due to thrombosis of other precerebral artery \| \| I63.10 \| Cerebral infarction due to embolism of unspecified precerebral artery \| \| I63.111 \| Cerebral infarction due to embolism of right vertebral artery \| \| I63.112 \| Cerebral infarction due to embolism of left vertebral artery \| \| I63.113 \| Cerebral infarction due to embolism of bilateral vertebral arteries \| \| I63.119 \| Cerebral infarction due to embolism of unspecified vertebral artery \| \| I63.12 \| Cerebral infarction due to embolism of basilar artery \| \| I63.131 \| Cerebral infarction due to embolism of right carotid artery \| \| I63.132 \| Cerebral infarction due to embolism of left carotid artery \| \| I63.133 \| Cerebral infarction due to embolism of bilateral carotid arteries \| \| I63.139 \| Cerebral infarction due to embolism of unspecified carotid artery \| \| I63.19 \| Cerebral infarction due to embolism of other precerebral artery \| \| I63.20 \| Cerebral infarction due to unspecified occlusion or stenosis of unspecified precerebral arteries \| \| I63.211 \| Cerebral infarction due to unspecified occlusion or stenosis of right vertebral artery \| \| I63.212 \| Cerebral infarction due to unspecified occlusion or stenosis of left vertebral artery \| \| I63.213 \| Cerebral infarction due to unspecified occlusion or stenosis of bilateral vertebral arteries \| \| I63.219 \| Cerebral infarction due to unspecified occlusion or stenosis of unspecified vertebral artery \| \| I63.22 \| Cerebral infarction due to unspecified occlusion or stenosis of basilar artery \| \| I63.231 \| Cerebral infarction due to unspecified occlusion or stenosis of right carotid arteries \| \| I63.232 \| Cerebral infarction due to unspecified occlusion or stenosis of left carotid arteries \| \| I63.233 \| Cerebral infarction due to unspecified occlusion or stenosis of bilateral carotid arteries \| \| I63.239 \| Cerebral infarction due to unspecified occlusion or stenosis of unspecified carotid artery \| \| I63.29 \| Cerebral infarction due to unspecified occlusion or stenosis of other precerebral arteries \| \| I63.30 \| Cerebral infarction due to thrombosis of unspecified cerebral artery \| \| I63.311 \| Cerebral infarction due to thrombosis of right middle cerebral artery \| \| I63.312 \| Cerebral infarction due to thrombosis of left middle cerebral artery \| \| I63.313 \| Cerebral infarction due to thrombosis of bilateral middle cerebral arteries \| \| I63.319 \| Cerebral infarction due to thrombosis of unspecified middle cerebral artery \| \| I63.321 \| Cerebral infarction due to thrombosis of right anterior cerebral artery \| \| I63.322 \| Cerebral infarction due to thrombosis of left anterior cerebral artery \| \| I63.323 \| Cerebral infarction due to thrombosis of bilateral anterior cerebral arteries \| \| I63.329 \| Cerebral infarction due to thrombosis of unspecified anterior cerebral artery \| \| I63.331 \| Cerebral infarction due to thrombosis of right posterior cerebral artery \| \| I63.332 \| Cerebral infarction due to thrombosis of left posterior cerebral artery \| \| I63.333 \| Cerebral infarction to thrombosis of bilateral posterior cerebral arteries \| \| I63.339 \| Cerebral infarction due to thrombosis of unspecified posterior cerebral artery \| \| I63.341 \| Cerebral infarction due to thrombosis of right cerebellar artery \| \| I63.342 \| Cerebral infarction due to thrombosis of left cerebellar artery \| \| I63.343 \| Cerebral infarction to thrombosis of bilateral cerebellar arteries \| \| I63.349 \| Cerebral infarction due to thrombosis of unspecified cerebellar artery \| \| I63.39 \| Cerebral infarction due to thrombosis of other cerebral artery \| \| I63.40 \| Cerebral infarction due to embolism of unspecified cerebral artery \| \| I63.411 \| Cerebral infarction due to embolism of right middle cerebral artery \| \| I63.412 \| Cerebral infarction due to embolism of left middle cerebral artery \| \| I63.413 \| Cerebral infarction due to embolism of bilateral middle cerebral arteries \| \| I63.419 \| Cerebral infarction due to embolism of unspecified middle cerebral artery \| \| I63.421 \| Cerebral infarction due to embolism of right anterior cerebral artery \| \| I63.422 \| Cerebral infarction due to embolism of left anterior cerebral artery \| \| I63.423 \| Cerebral infarction due to embolism of bilateral anterior cerebral arteries \| \| I63.429 \| Cerebral infarction due to embolism of unspecified anterior cerebral artery \| \| I63.431 \| Cerebral infarction due to embolism of right posterior cerebral artery \| \| I63.432 \| Cerebral infarction due to embolism of left posterior cerebral artery \| \| I63.433 \| Cerebral infarction due to embolism of bilateral posterior cerebral arteries \| \| I63.439 \| Cerebral infarction due to embolism of unspecified posterior cerebral artery \| \| I63.441 \| Cerebral infarction due to embolism of right cerebellar artery \| \| I63.442 \| Cerebral infarction due to embolism of left cerebellar artery \| \| I63.443 \| Cerebral infarction due to embolism of bilateral cerebellar arteries \| \| I63.449 \| Cerebral infarction due to embolism of unspecified cerebellar artery \| \| I63.49 \| Cerebral infarction due to embolism of other cerebral artery \| \| I63.50 \| Cerebral infarction due to unspecified occlusion or stenosis of unspecified cerebral artery \| \| I63.511 \| Cerebral infarction due to unspecified occlusion or stenosis of right middle cerebral artery \| \| I63.512 \| Cerebral infarction due to unspecified occlusion or stenosis of left middle cerebral artery \| \| I63.513 \| Cerebral infarction due to unspecified occlusion or stenosis of bilateral middle cerebral arteries \| \| I63.519 \| Cerebral infarction due to unspecified occlusion or stenosis of unspecified middle cerebral artery \| \| I63.521 \| Cerebral infarction due to unspecified occlusion or stenosis of right anterior cerebral artery \| \| I63.522 \| Cerebral infarction due to unspecified occlusion or stenosis of left anterior cerebral artery \| \| I63.523 \| Cerebral infarction due to unspecified occlusion or stenosis of bilateral anterior cerebral arteries \| \| I63.529 \| Cerebral infarction due to unspecified occlusion or stenosis of unspecified anterior cerebral artery \| \| I63.531 \| Cerebral infarction due to unspecified occlusion or stenosis of right posterior cerebral artery \| \| I63.532 \| Cerebral infarction due to unspecified occlusion or stenosis of left posterior cerebral artery \| \| I63.533 \| Cerebral infarction due to unspecified occlusion or stenosis of bilateral posterior cerebral arteries \| \| I63.539 \| Cerebral infarction due to unspecified occlusion or stenosis of unspecified posterior cerebral artery \| \| I63.541 \| Cerebral infarction due to unspecified occlusion or stenosis of right cerebellar artery \| \| I63.542 \| Cerebral infarction due to unspecified occlusion or stenosis of left cerebellar artery \| \| I63.543 \| Cerebral infarction due to unspecified occlusion or stenosis of bilateral cerebellar arteries \| \| I63.549 \| Cerebral infarction due to unspecified occlusion or stenosis of unspecified cerebellar artery \| \| I63.59 \| Cerebral infarction due to unspecified occlusion or stenosis of other cerebral artery \| \| I63.6 \| Cerebral infarction due to cerebral venous thrombosis, nonpyogenic \| \| I63.8 \| Other cerebral infarction \| \| I63.81 \| Other cerebral infarction due to occlusion or stenosis of small artery \| \| I63.89 \| Other cerebral infarction \| \| I63.9 \| Cerebral infarction, unspecified \| \| I65.01 \| Occlusion and stenosis of right vertebral artery \| \| I65.02 \| Occlusion and stenosis of left vertebral artery \| \| I65.03 \| Occlusion and stenosis of bilateral vertebral arteries \| \| I65.09 \| Occlusion and stenosis of unspecified vertebral artery \| \| I65.1 \| Occlusion and stenosis of basilar artery \| \| I65.21 \| Occlusion and stenosis of right carotid artery \| \| I65.22 \| Occlusion and stenosis of left carotid artery \| \| I65.23 \| Occlusion and stenosis of bilateral carotid arteries \| \| I65.29 \| Occlusion and stenosis of unspecified carotid artery \| \| I65.8 \| Occlusion and stenosis of other precerebral arteries \| \| I65.9 \| Occlusion and stenosis of unspecified precerebral artery \| \| I67.0 \| Dissection of cerebral arteries, nonruptured \| \| I67.1 \| Cerebral aneurysm, nonruptured \| \| I67.2 \| Cerebral atherosclerosis \| \| I67.3 \| Progressive vascular leukoencephalopathy \| \| I67.4 \| Hypertensive encephalopathy \| \| I67.5 \| Moyamoya disease \| \| I67.6 \| Nonpyogenic thrombosis of intracranial venous system \| \| I67.7 \| Cerebral arteritis, not elsewhere classified \| \| I67.81 \| Acute cerebrovascular insufficiency \| \| I67.82 \| Cerebral ischemia \| \| I67.83 \| Posterior reversible encephalopathy syndrome \| \| I67.841 \| Reversible cerebrovascular vasoconstriction syndrome \| \| I67.848 \| Other cerebrovascular vasospasm and vasoconstriction \| \| I67.850 \| Cerebral autosomal dominant arteriopathy with subcortical infarcts and leukoencephalopathy \| \| I67.858 \| Other hereditary cerebrovascular disease \| \| I67.89 \| Other cerebrovascular disease \| \| I67.9 \| Cerebrovascular disease, unspecified \| \| I68.8 \| Other cerebrovascular disorders in diseases classified elsewhere \| \| I69.30 \| Unspecified sequelae of cerebral infarction \| \| I69.31 \| Cognitive Deficits following Cerebral Infarction \| \| I69.310 \| Attention and concentration deficit following cerebral infarction \| \| I69.311 \| Memory deficit following cerebral infarction \| \| I69.312 \| Visuospatial deficit and spatial neglect following cerebral infarction \| \| I69.313 \| Psychomotor deficit following cerebral infarction \| \| I69.314 \| Frontal lobe and executive function deficit following cerebral infarction \| \| I69.315 \| Cognitive social or emotional deficit following cerebral infarction \| \| I69.318 \| Other symptoms and signs involving cognitive functions following cerebral infarction \| \| I69.319 \| Unspecified symptoms and signs involving cognitive functions following cerebral infarction \| \| I69.320 \| Aphasia following cerebral infarction \| \| I69.321 \| Dysphasia following cerebral infarction \| \| I69.322 \| Dysarthria following cerebral infarction \| \| I69.323 \| Fluency disorder following cerebral infarction \| \| I69.328 \| Other speech and language deficits following cerebral infarction \| \| I69.331 \| Monoplegia of upper limb following cerebral infarction affecting right dominant side \| \| I69.332 \| Monoplegia of upper limb following cerebral infarction affecting left dominant side \| \| I69.333 \| Monoplegia of upper limb following cerebral infarction affecting right non-dominant side \| \| I69.334 \| Monoplegia of upper limb following cerebral infarction affecting left non-dominant side \| \| I69.339 \| Monoplegia of upper limb following cerebral infarction affecting unspecified side \| \| I69.341 \| Monoplegia of lower limb following cerebral infarction affecting right dominant side \| \| I69.342 \| Monoplegia of lower limb following cerebral infarction affecting left dominant side \| \| I69.343 \| Monoplegia of lower limb following cerebral infarction affecting right non-dominant side \| \| I69.344 \| Monoplegia of lower limb following cerebral infarction affecting left non-dominant side \| \| I69.349 \| Monoplegia of lower limb following cerebral infarction affecting unspecified side \| \| I69.351 \| Hemiplegia and hemiparesis following cerebral infarction affecting right dominant side \| \| I69.352 \| Hemiplegia and hemiparesis following cerebral infarction affecting left dominant side \| \| I69.353 \| Hemiplegia and hemiparesis following cerebral infarction affecting right non-dominant side \| \| I69.354 \| Hemiplegia and hemiparesis following cerebral infarction affecting left non-dominant side \| \| I69.359 \| Hemiplegia and hemiparesis following cerebral infarction affecting unspecified side \| \| I69.361 \| Other paralytic syndrome following cerebral infarction affecting right dominant side \| \| I69.362 \| Other paralytic syndrome following cerebral infarction affecting left dominant side \| \| I69.363 \| Other paralytic syndrome following cerebral infarction affecting right non-dominant side \| \| I69.364 \| Other paralytic syndrome following cerebral infarction affecting left non-dominant side \| \| I69.365 \| Other paralytic syndrome following cerebral infarction, bilateral \| \| I69.369 \| Other paralytic syndrome following cerebral infarction affecting unspecified side \| \| I69.390 \| Apraxia following cerebral infarction \| \| I69.391 \| Dysphagia following cerebral infarction \| \| I69.392 \| Facial weakness following cerebral infarction \| \| I69.393 \| Ataxia following cerebral infarction \| \| I69.398 \| Other sequelae of cerebral infarction \| \| I69.80 \| Unspecified sequelae of other cerebrovascular disease \| \| I69.81 \| Cognitive Deficits following other Cerebrovascular Disease \| \| I69.810 \| Attention and concentration deficit following other cerebrovascular disease \| \| I69.811 \| Memory deficit following other cerebrovascular disease \| \| I69.812 \| Visuospatial deficit and spatial neglect following other cerebrovascular disease \| \| I69.813 \| Psychomotor deficit following other cerebrovascular disease \| \| I69.814 \| Frontal lobe and executive function deficit following other cerebrovascular disease \| \| I69.815 \| Cognitive social or emotional deficit following other cerebrovascular disease \| \| I69.818 \| Other symptoms and signs involving cognitive functions following other cerebrovascular disease \| \| I69.819 \| Unspecified symptoms and signs involving cognitive functions following other cerebrovascular disease \| \| I69.820 \| Aphasia following other cerebrovascular disease \| \| I69.821 \| Dysphasia following other cerebrovascular disease \| \| I69.822 \| Dysarthria following other cerebrovascular disease \| \| I69.823 \| Fluency disorder following other cerebrovascular disease \| \| I69.828 \| Other speech and language deficits following other cerebrovascular disease \| \| I69.831 \| Monoplegia of upper limb following other cerebrovascular disease affecting right dominant side \| \| I69.832 \| Monoplegia of upper limb following other cerebrovascular disease affecting left dominant side \| \| I69.833 \| Monoplegia of upper limb following other cerebrovascular disease affecting right non-dominant side \| \| I69.834 \| Monoplegia of upper limb following other cerebrovascular disease affecting left non-dominant side \| \| I69.839 \| Monoplegia of upper limb following other cerebrovascular disease affecting unspecified side \| \| I69.841 \| Monoplegia of lower limb following other cerebrovascular disease affecting right dominant side \| \| I69.842 \| Monoplegia of lower limb following other cerebrovascular disease affecting left dominant side \| \| I69.843 \| Monoplegia of lower limb following other cerebrovascular disease affecting right non-dominant side \| \| I69.844 \| Monoplegia of lower limb following other cerebrovascular disease affecting left non-dominant side \| \| I69.849 \| Monoplegia of lower limb following other cerebrovascular disease affecting unspecified side \| \| I69.851 \| Hemiplegia and hemiparesis following other cerebrovascular disease affecting right dominant side \| \| I69.852 \| Hemiplegia and hemiparesis following other cerebrovascular disease affecting left dominant side \| \| I69.853 \| Hemiplegia and hemiparesis following other cerebrovascular disease affecting right non-dominant side \| \| I69.854 \| Hemiplegia and hemiparesis following other cerebrovascular disease affecting left non-dominant side \| \| I69.859 \| Hemiplegia and hemiparesis following other cerebrovascular disease affecting unspecified side \| \| I69.861 \| Other paralytic syndrome following other cerebrovascular disease affecting right dominant side \| \| I69.862 \| Other paralytic syndrome following other cerebrovascular disease affecting left dominant side \| \| I69.863 \| Other paralytic syndrome following other cerebrovascular disease affecting right non-dominant side \| \| I69.864 \| Other paralytic syndrome following other cerebrovascular disease affecting left non-dominant side \| \| I69.865 \| Other paralytic syndrome following other cerebrovascular disease, bilateral \| \| I69.869 \| Other paralytic syndrome following other cerebrovascular disease affecting unspecified side \| \| I69.890 \| Apraxia following other cerebrovascular disease \| \| I69.891 \| Dysphagia following other cerebrovascular disease \| \| I69.892 \| Facial weakness following other cerebrovascular disease \| \| I69.893 \| Ataxia following other cerebrovascular disease \| \| I69.898 \| Other sequelae of other cerebrovascular disease \| \| I69.90 \| Unspecified sequelae of unspecified cerebrovascular disease \| \| I69.91 \| Cognitive Deficits following unspecified Cerebrovascular Disease \| \| I69.910 \| Attention and concentration deficit following unspecified cerebrovascular disease \| \| I69.911 \| Memory deficit following unspecified cerebrovascular disease \| \| I69.912 \| Visuospatial deficit and spatial neglect following unspecified cerebrovascular disease \| \| I69.913 \| Psychomotor deficit following unspecified cerebrovascular disease \| \| I69.914 \| Frontal lobe and executive function deficit following unspecified cerebrovascular disease \| \| I69.915 \| Cognitive social or emotional deficit following unspecified cerebrovascular disease \| \| I69.918 \| Other symptoms and signs involving cognitive functions following unspecified cerebrovascular disease \| \| I69.919 \| Unspecified symptoms and signs involving cognitive functions following unspecified cerebrovascular disease \| \| I69.920 \| Aphasia following unspecified cerebrovascular disease \| \| I69.921 \| Dysphasia following unspecified cerebrovascular disease \| \| I69.922 \| Dysarthria following unspecified cerebrovascular disease \| \| I69.923 \| Fluency disorder following unspecified cerebrovascular disease \| \| I69.928 \| Other speech and language deficits following unspecified cerebrovascular disease \| \| I69.931 \| Monoplegia of upper limb following unspecified cerebrovascular disease affecting right dominant side \| \| I69.932 \| Monoplegia of upper limb following unspecified cerebrovascular disease affecting left dominant side \| \| I69.933 \| Monoplegia of upper limb following unspecified cerebrovascular disease affecting right non-dominant side \| \| I69.934 \| Monoplegia of upper limb following unspecified cerebrovascular disease affecting left non-dominant side \| \| I69.939 \| Monoplegia of upper limb following unspecified cerebrovascular disease affecting unspecified side \| \| I69.941 \| Monoplegia of lower limb following unspecified cerebrovascular disease affecting right dominant side \| \| I69.942 \| Monoplegia of lower limb following unspecified cerebrovascular disease affecting left dominant side \| \| I69.943 \| Monoplegia of lower limb following unspecified cerebrovascular disease affecting right non-dominant side \| \| I69.944 \| Monoplegia of lower limb following unspecified cerebrovascular disease affecting left non-dominant side \| \| I69.949 \| Monoplegia of lower limb following unspecified cerebrovascular disease affecting unspecified side \| \| I69.951 \| Hemiplegia and hemiparesis following unspecified cerebrovascular disease affecting right dominant side \| \| I69.952 \| Hemiplegia and hemiparesis following unspecified cerebrovascular disease affecting left dominant side \| \| I69.953 \| Hemiplegia and hemiparesis following unspecified cerebrovascular disease affecting right non-dominant side \| \| I69.954 \| Hemiplegia and hemiparesis following unspecified cerebrovascular disease affecting left non-dominant side \| \| I69.959 \| Hemiplegia and hemiparesis following unspecified cerebrovascular disease affecting unspecified side \| \| I69.961 \| Other paralytic syndrome following unspecified cerebrovascular disease affecting right dominant side \| \| I69.962 \| Other paralytic syndrome following unspecified cerebrovascular disease affecting left dominant side \| \| I69.963 \| Other paralytic syndrome following unspecified cerebrovascular disease affecting right non-dominant side \| \| I69.964 \| Other paralytic syndrome following unspecified cerebrovascular disease affecting left non-dominant side \| \| I69.965 \| Other paralytic syndrome following unspecified cerebrovascular disease, bilateral \| \| I69.969 \| Other paralytic syndrome following unspecified cerebrovascular disease affecting unspecified side \| \| I69.990 \| Apraxia following unspecified cerebrovascular disease \| \| I69.991 \| Dysphagia following unspecified cerebrovascular disease \| \| I69.992 \| Facial weakness following unspecified cerebrovascular disease \| \| I69.993 \| Ataxia following unspecified cerebrovascular disease \| \| I69.998 \| Other sequelae following unspecified cerebrovascular disease \| \| Z86.73 \| Personal history of transient ischemic attack (TIA), and cerebral infarction without residual deficits \| |

**Supplemental Table 2. Adjusted Hazards Ratios for Mortality for the Interaction between Race and Ethnicity, and Incident Stroke**

|  | **Adjusted Hazard Ratios (95% CI)** | |
| --- | --- | --- |
| **Race and Ethnicity** | **With Stroke** | **Without Stroke** |
| American Indian / Alaska Native |  |  |
| Asian | 1.11 (0.84-1.48) | 0.91 (0.79-1.04) |
| Black | 1.48 (1.34-1.62) | 0.90 (0.87-0.94) |
| Hispanic | 1.40 (1.22-1.63) | 0.80 (0.75-0.84) |
| Multiracial | 2.21 (1.54-3.17) | 0.93 (0.81-1.06) |
| White | 1.37 (1.32-1.41) | Reference |
